# Vim-Thalamic Deep Brain Stimulation for Cervical Dystonia and Upper-Limb Tremor: Quantification by Markerless-3D Kinematics and Accelerometry

**DOI:** 10.5334/tohm.673

**Published:** 2022-03-10

**Authors:** Xenos L. Mason, Katy A. Cross, Ahmet Arac, Yvette Bordelon, Allan D. Wu

**Affiliations:** 1 Departments of Neurological Surgery and Neurology, University of Southern California Keck School of Medicine, Los Angeles, CA, US; 2 Department of Neurology, University of California Los Angeles David Geffen School of Medicine, Los Angeles, California, US; 3 Department of Neurology, Northwestern University Feinberg School of Medicine, Chicago, Illinois, US

**Keywords:** Deep Brain Stimulation, Dystonia, Tremor, Accelerometry, Kinematics, Ventral Thalamic Nuclei

## Abstract

**Background:**

Deep Brain Stimulation (DBS) for dystonia is usually targeted to the globus pallidus internus (GPi), though stimulation of the ventral-intermediate nucleus of the thalamus (Vim) can be an effective treatment for phasic components of dystonia including tremor. We report on a patient who developed a syndrome of bilateral upper limb postural and action tremor and progressive cervical dystonia with both phasic and tonic components which were responsive to Vim DBS. We characterize and quantify this effect using markerless-3D-kinematics combined with accelerometry.

**Methods:**

Stereo videography was used to record our subject in 3D. The DeepBehavior toolbox was applied to obtain timeseries of joint position for kinematic analysis [1]. Accelerometry was performed simultaneously for comparison with prior literature.

**Results:**

Bilateral Vim DBS improved both dystonic tremor magnitude and tonic posturing. DBS of the hemisphere contralateral to the direction of dystonic head rotation (left Vim) had greater efficacy. Assessment of tremor magnitude by 3D-kinematics was concordant with accelerometry and was able to quantify tonic dystonic posturing.

**Discussion:**

In this case, Vim DBS treated both cervical dystonic tremor and dystonic posturing. Markerless-3D-kinematics should be further studied as a method of quantifying and characterizing tremor and dystonia.

## Introduction

Essential tremor (ET) is one of the most common movement disorders, with an overall population prevalence of approximately one percent [2]. In patients who do not obtain adequate benefit from first-line pharmacotherapy alone or cannot tolerate side-effects, deep brain stimulation targeting the ventral intermediate nucleus of the thalamus (Vim DBS) can provide effective control of tremor [2,3]. DBS of the globus pallidus internus (GPi) is a well-established and effective treatment for dystonia. However Vim lesioning and neuromodulation have also been used [4]. In general, Vim DBS robustly improves dystonic tremor but is less effective for dystonic posturing [5,6].

Accelerometry is a well-established method of characterizing and quantifying tremor and has been used to differentiate oscillatory and irregular components of phasic dystonic movements [7]. Although accelerometry cannot itself detect tonic dystonic posturing, this can sometimes be inferred by the dependence of phasic movements (i.e. tremor) on head position (based on position with least tremor, i.e. *null* point), and the presence of associated non-oscillatory (“jerky”) phasic movements that reflect an asymmetric tendency towards the dystonic posture [8]. Video-based markerless-3D kinematic analysis has not to our knowledge been used to assess tremor. Compared to accelerometry, kinematics has the added advantage of directly measuring joint position to quantify tonic dystonic movements (posturing).

### Subject Case Description

A 56-year-old right-handed man with a thirty-year history of upper limb postural/action tremor (diagnosed as ET) was seen at our center. He had been prescribed propranolol, primidone, and valium, but was intolerant of these medications due to side-effects. Six years prior to presentation, he had undergone implantation of left Vim DBS for medication-refractory dominant right-arm tremor. A post-operative CT scan showed appropriate placement of the electrode without associated hemorrhage or infarct (supp. fig. 1a i–iii). Three years later, he developed cervical dystonia with rightward tilt, rotation, and head tremor. He noted improvement in his tremor when gently holding his chin (sensory trick). Two years later he underwent implantation of right Vim DBS for worsening tremor in the left arm. An examination at the time of initial right Vim programming noted unchanged cervical dystonia, and monopolar stimulation was established (Case +, Contact 9 -). Two months later he reported worsening neck pulling, which partially but immediately improved after DBS reprogramming. Settings at that time were: (Left-Vim) (interleaved) Case +, Contact 1 –, 3.3 V, 150 *u*s, 125 Hz/Case +, Contact 2 –, 3.7 V, 140 *u*s, 125 Hz; (Right-Vim) (interleaved) Case +, Contact 9 –, 3.4 V, 150 *u*s, 125 Hz/Case +, Contact 11 –, 1.8 V, 110 *u*s, 125 Hz. He was treated with escalating doses of onabotulinumtoxin A (Botox) injection to the left sternocleidomastoid (up to 75 units), right levator scapulae (up to 20 units), and later the bilateral splenius capitis (up to 120 units on the right and 40 units on the left) with only partial improvement.

He had a medical history notable for hypertension and type-II diabetes. His medications included glimepiride and lisinopril. He denied any prior history of frequent cramping or abnormal posturing of the arms or neck, head tremor or turning, or writer’s cramp. There was no family history of tremor. He did not drink alcohol. Our initial examination off-stimulation showed severe postural and action tremor of the upper extremities (left more than right), cervical dystonia (rightward rotation and rightward tilt), and a high-amplitude, irregular, multi-direction tremor of the head. On presentation, activation of right Vim DBS provided a small degree of improvement in dystonia and head tremor; upon activation of left Vim DBS, he was able to return his head to a midline position, and his head tremor resolved. A monopolar review (conducted at the nadir of botulinum toxin efficacy, just prior to injection) demonstrated immediate improvement in dystonia and dystonic tremor from ventral contact stimulation. This effect was small from right Vim DBS, but much more apparent from left Vim DBS at similar voltages, with the greatest subjective improvement from bilateral stimulation. In general, his limb tremor improved at lower voltages than did his cervical dystonia. There was no difference in left Vim threshold voltages (for detectable efficacy or side-effects) with the right Vim either ON or OFF. His stimulation was optimized with the following settings: (Left-Vim) Case +, Contact 1 –, 4.2 V, 70 *u*s, 150 Hz; (Right-Vim) Case +, Contact 9 –, 3.4 V, 130 *u*s, 125 Hz.

## Methods

Informed consent was obtained from the patient for publication of case details and images. The following protocol was performed approximately two hours after botulinum toxin injection (prior to onset of action and three months after prior injection). Using a protractor-goniometer as a guide, the patient was instructed to rotate his head to specific angles (midline, 20 and 40 degrees from midline in both left and right directions). 20 seconds of simultaneous accelerometry and markerless-3D-kinematics were obtained at each position. 3D-kinematics data was obtained with a stereo video recording system at 170 frames per second as previously described [1]. Accelerometry data was obtained by an iPhoneXS (Apple Inc.) affixed to the lateral aspect of the head at the right temple, running the “Vibration” application (Diffraction Limited Design LLC, sampling rate 50 Hz). This process was repeated with either left, right, or bilateral Vim DBS activation to previously optimized parameters.

**Figure 1:**
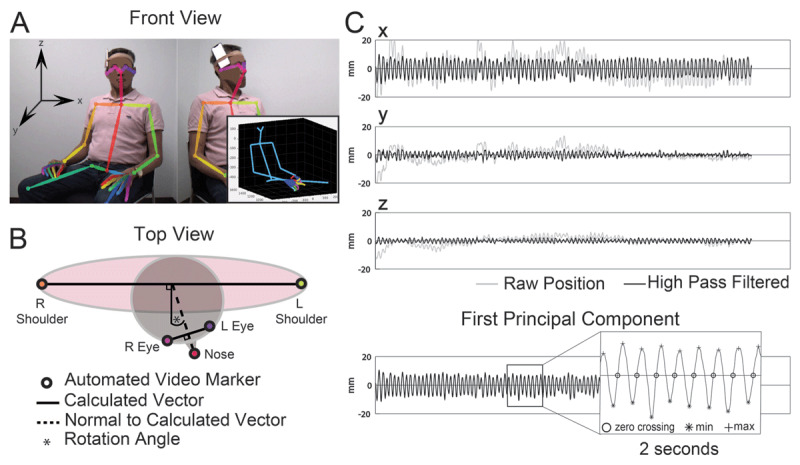
**Illustration of methods. (A)** Example images from simultaneous stereo videography recording, with automated virtual markers applied by the DeepBehavior toolbox. Axes illustrated apply to both 3D kinematic and accelerometric analysis. Inset: Skeletal 3D reconstruction based on automated markers **(B)** Vectors connecting the L and R shoulders and L and R eyes were used to calculate the head rotation angle, relative to forward-facing. Tonic head position was then quantified as the average of the head rotation angle over the entire 20-second trial. (**C**) Tremor was quantified using the position of the face in 3D space over time (average of L eye, R eye, and nose marker positions). To remove drift and isolate oscillatory movement, face position was high-pass filtered (light gray line: unfiltered). Principal component analysis was applied to be agnostic to movement direction in identifying tremor cycles. Tremor frequency was obtained by deriving cycle length via an average of time between zero-crossings. The path-length (distance travelled) of one virtual marker during one cycle length (time) was used as a measure of tremor magnitude.

The DeepBehavior toolbox was used to extract timeseries of 3D joint positions from stereo video (***Figure 1a***) using a previously trained neural network [1]. Head rotation angle was calculated at each time point based on the position of the eyes relative to the shoulders (***Figure 1b***). The head rotation angle was averaged over the entire 20-seconds to quantify tonic posture. Tremor magnitude was calculated as the average distance traveled by the face for each tremor cycle, defined as follows. First, a time series of face position in 3D space (X, Y, Z coordinates) was calculated as the average of eye and nose positions, which served to reduce measurement noise. The face position time series was high-pass filtered with 2 Hz cutoff with a zero-phase fourth-order Butterworth filter to remove drift (***Figure 1c***). The first principal component of X, Y and Z face position was obtained to provide a direction invariant representation of the tremor to define tremor cycles (***Figure 1c***, inset). Tremor cycle onsets were identified as the time of positive-going-zero crossings of the first principal component, limited to only those data segments including a minimum of 3 consecutive cycles of similar frequency (<2 Hz difference, thereby defining oscillatory tremor [9]). Tremor magnitude was then calculated as the average of the distance travelled by the face (sum of Euclidean distance between consecutive X, Y, Z coordinates) for each tremor cycle.

For accelerometric data, the axis of greatest tremor amplitude was used to approximate total tremor amplitude (x-axis, corresponding to L/R head rotation, in units of m/s^2^). Acceleration was converted to displacement (units of mm) using the equation for a simple harmonic oscillator (*A* = –(2*πf*)^2^ *d*) omitting the negative sign to account for the physiology of forces in the direction of movement [10]. Frequency was estimated as the single peak frequency derived from a Fast Fourier transform (performed by the “Vibration” application) of the x-axis displacement.

The left DBS electrode was localized using the processing pipeline in Lead-DBS (lead-dbs.org) [11]. Briefly the postoperative CT was co-registered to the preoperative MRI using a two-stage linear registration as implemented in Advanced Normalization Tools [12]. Pre- and post-operative acquisitions were spatially normalized into ICBM 2009b NLIN asymmetric (Montreal Neurologic Institute [MNI]) space based on preoperative acquisitions using the Unified Segmentation Approach as implemented in SPM12 [13]. Electrode contacts were automatically pre-reconstructed using the TRAC/CORE approach and manually refined using a tool specifically designed for this task in Lead-DBS [11]. 3D electrode reconstructions were rendered in Lead-DBS using anatomic segmentation defined by the DISTAL Atlas [14].

## Results

**Figure 2:**
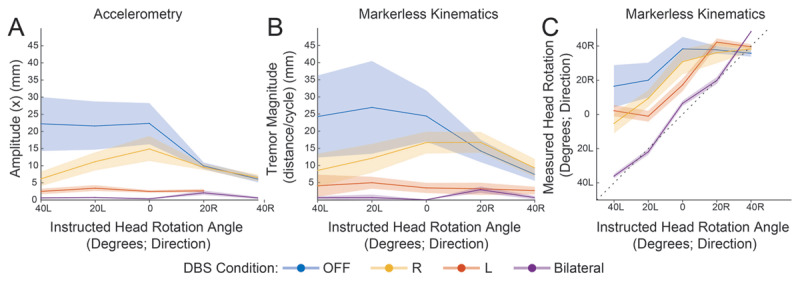
**Bilateral Vim-DBS additively reduces phasic and tonic (posturing) components of cervical dystonia.** A tremor “null-point” is suggested beyond the 40-degree rightward rotation. Plots depict average value over the duration of each trial and shading represents the standard deviation. (**A**) Accelerometry in the axis of greatest tremor amplitude demonstrates tremor reductions in all directions of head rotation from left Vim-DBS (orange, note missing data at 40R), right Vim-DBS (yellow, less effective), and bilateral Vim-DBS (purple). (**B**) Markerless-3D-kinematic analysis demonstrates similar findings. (**C**) Markerless-3D-kinematic analysis demonstrates improvement in tonic dystonia (posturing). With DBS OFF, the patient is unable to comply with requested head rotation and maintains a rightward rotation throughout all conditions. With bilateral Vim-DBS activation the patient has near-perfect control of head rotation (illustrated by the dotted line). Right and left Vim-DBS are each partially effective in facilitating head rotation.

Bilateral Vim DBS reduced the amplitude of dystonic head tremor (averaged across all head positions) as measured by accelerometry (amplitude (mm): DBS OFF: 16.3 SD = 11.8; DBS Right: 9.5 SD = 5.1; DBS Left 2.9 SD = 1.4; DBS Bilateral 0.7 SD = 0.9) (***Figure 2b***) and markerless-3D-kinematics (DBS OFF: 17.0 SD = 6.8; DBS Right: 11.0 SD = 3.6; DBS Left 2.8 SD = 0.7; DBS Bilateral 1.0 SD = 1.0) (***Figure 2b***). Both methods of quantification similarly showed a higher effect size from left Vim stimulation (***Figure 2a, 2b***). Markerless-3D-kinematics also demonstrated that bilateral Vim DBS reduced tonic cervical dystonia (posturing), indicated by a decreased “head-rotation angle error” defined as the absolute value of [instructed head rotation angle]-[actual head rotation angle]: (mean error (degrees)) DBS OFF: 31.4° SD = 20.5; DBS Right 22.5° SD = 13.6; DBS Left: 20.2° SD = 14.9; DBS Bilateral 4.1° SD = 3.3) (***Figure 2c***). DBS condition had no effect on peak tremor frequency (supp. fig. 2). Tremor frequency was equivalent when calculated using spectral analysis or the inverse of average cycle duration of the first principal component. Accelerometry of the upper extremity tremor with DBS OFF revealed a regular postural tremor with a sharp peak frequency at 3.7 Hz (supp. fig. 2). Localization of the left Vim electrode demonstrated placement in the medial Vim at the border of the internal and external subnuclei (supp. fig. 1b, 1c). A post-operative CT scan was not available for localization of the right Vim electrode.

## Discussion

Video-based markerless-3D-kinematic analysis produces comparable results to accelerometry in analysis of peak tremor frequency, power spectra, and tremor magnitude. We do not make a direct comparison between accelerometry and markerless-3D-kinematics in the measurement of tonic dystonia. However, the reduction in tremor amplitude with rightward head rotation as measured by accelerometry (***Figure 2a***) suggests a tremor-null point in the direction of tonic dystonic head rotation. The loss of this relationship between head-rotation and tremor amplitude during bilateral Vim DBS (***Figure 2a, 2b***) suggests a decrease in tonic dystonia, which is consistent with the joint-position measurements by markerless-3D-kinematics (***Figure 2c***). In the future, this technology could facilitate analysis of complex and irregular tremors (i.e. rubral tremor, dystonic tremor) without sacrificing the fidelity of accelerometry. An additional advantage is the ability to directly measure joint position, and thereby simultaneously assess both phasic (tremulous, jerky), and tonic (fixed posturing) components of dystonia. Further studies designed to validate this method against gold-standard accelerometry are warranted. Compared to typical motion capture systems, this markerless approach uses a portable camera setup that is more conducive to clinical use, does not require application of markers or devices to the patient, and has low cost.

An important caveat to our case description is the difficulty in assigning a diagnosis of ET, versus whether alternatively this patient’s tremor was a manifestation of dystonia. On one hand, dystonia has a higher prevalence in patients with ET (now sometimes controversially called “ET+Dystonia”) [15]. Findings of the ET study group suggest the prevalence of coincident dystonia is 2.9–10.2% [15]. An alternative explanation is that our patient had, in fact, longstanding upper-limb “tremor associated with dystonia” (TAWD, defined as tremor in body segments uninvolved by dystonia) and that overt cervical dystonia did not develop (or was asymptomatic) for many years. Postural/kinetic upper-limb tremor is common in patients with cervical dystonia (10–25% incidence), and indeed may precede onset of dystonia by years or even decades [15,16,17]. EMG characteristics of ET and TAWD (such as tremor frequency, burst morphology, and weight-loading effects) do not differ in most patients, though TAWD may have a more variable frequency [9,18].

In this case, initial unilateral Vim DBS implantation was associated with delayed development of cervical dystonia. Dystonia can occur after thalamic stroke, and infarcts involving the external/internal Vim have been shown to be strongly associated with secondary dystonia [19]. However, dystonia after thalamotomy (thermo-ablative, radiofrequency, or MR-guided-focused-ultrasound) is very rare [4,20,21,22,23,24,25,26]. Two prior case series report worsening of pre-existing dystonic symptoms after Vim DBS implantation for tremor [5,27]. The time course of our case (dystonia onset following years after left Vim electrode implantation) and the absence of post-operative hemorrhage or infarct (supp. fig. 1a) would suggest that cervical dystonia was not a direct side-effect of Vim DBS implantation.

In our patient, Vim DBS immediately reduced both dystonic head tremor (phasic) and dystonic posturing (tonic), and bilateral stimulation was shown to be additively effective. Five single center retrospective studies have assessed the effect of Vim DBS on dystonic tremor and dystonia, and all have shown good efficacy for dystonic tremor but very little improvement in tonic dystonic posturing [5,28,29,30,31]. Indeed a 2006 metanalysis of 24 studies (including 137 patients) of DBS for dystonia demonstrated a modest improvement of only 10.6% in Burke-Fahn-Marsden dystonia rating scale after Vim DBS compared to 54% following Gpi DBS [6].

The rapid and near-complete improvement of our patient’s dystonic posturing with Vim DBS is difficult to reconcile with this retrospective literature. However, one prior report describes a similar case of Vim DBS-responsive upper-extremity tremor with concurrent improvement in cervical dystonia [32]. Two very recent cases describe similar improvement in tonic components of craniocervical dystonia after bilateral Vim DBS [33,34]. It is thus possible that in some patients with dystonia, the underlying “circuitopathy” is localized to the cerebellothalamic pathways, as has been suggested by functional and structural neuroimaging [35,36,37]. In these patients, a shared cerebellar pathophysiology may produce both dystonia and an ET-like upper limb postural and action tremor, which would then both respond to DBS of the cerebello-thalamic relay in the Vim.

The most significant limitation of our single-subject study is that we are not able to draw any statistical conclusion on the equivalence of markerless-3D-kinematics with gold-standard accelerometry. As such our aim was to illustrate the potential of this technology for future research. Vim-DBS was unusually efficacious for treatment of dystonia in this case, and our study does not offer insight into generalization: how to identify similar patients or define specific DBS programming associated with improvement. Further research of Vim-DBS for dystonia is warranted, including to identify biomarkers that predict response, and define optimal programming strategies. Subjective, patient-centered measures (such as global-impression of change) would also be an important accompaniment to objective data in a larger trial. Regarding technical limitations, time-stamped synchronization of videography and accelerometry data streams could allow for other ways to compare the two signal waveforms. Reconstruction of the volume-of-tissue-activation can offer important insight into DBS efficacy, especially for novel indications and targets. In spite of these limitations, the approach and data presented in this proof-of-concept study can inform future methodological research in quantification of tremor and dystonia, and generates testable hypotheses on the effect of thalamic stimulation for tonic dystonic posturing.

## Additonal File

The additonal file for this article can be found as follows:

10.5334/tohm.673.s1 Supplemental Figures. Figures 1–3.
