# Supplementary material for: Vim-Thalamic Deep Brain Stimulation for Cervical Dystonia and Upper-Limb Tremor: Quantification by Markerless-3D Kinematics and Accelerometry
Source: Tremor Other Hyperkinet Mov (N Y). 2022 Mar 10;12:5. doi: 10.5334/tohm.673 (PMC8916052; doi:10.5334/tohm.673)

**Supplemental Figure 1: Post-operative imaging and electrode localization. (A)** Ventral (i) to dorsal (iii) axial post-operative CT imaging following left-Vim DBS implantation shows the electrode in the posterolateral thalamus without associated hemorrhage or infarction. **(B, C)** 3D reconstruction of left DBS electrode localization from frontal **(B)** and oblique lateral **(C)** views, based on co-registration of preoperative MRI and post-operative CT imaging. The Vim (internal and external subnuclei) is shown in pale yellow, rendered in MNI space (shown as a greyscale brain image). Red boxes show an enlarged view.

Supplemental Figure 1

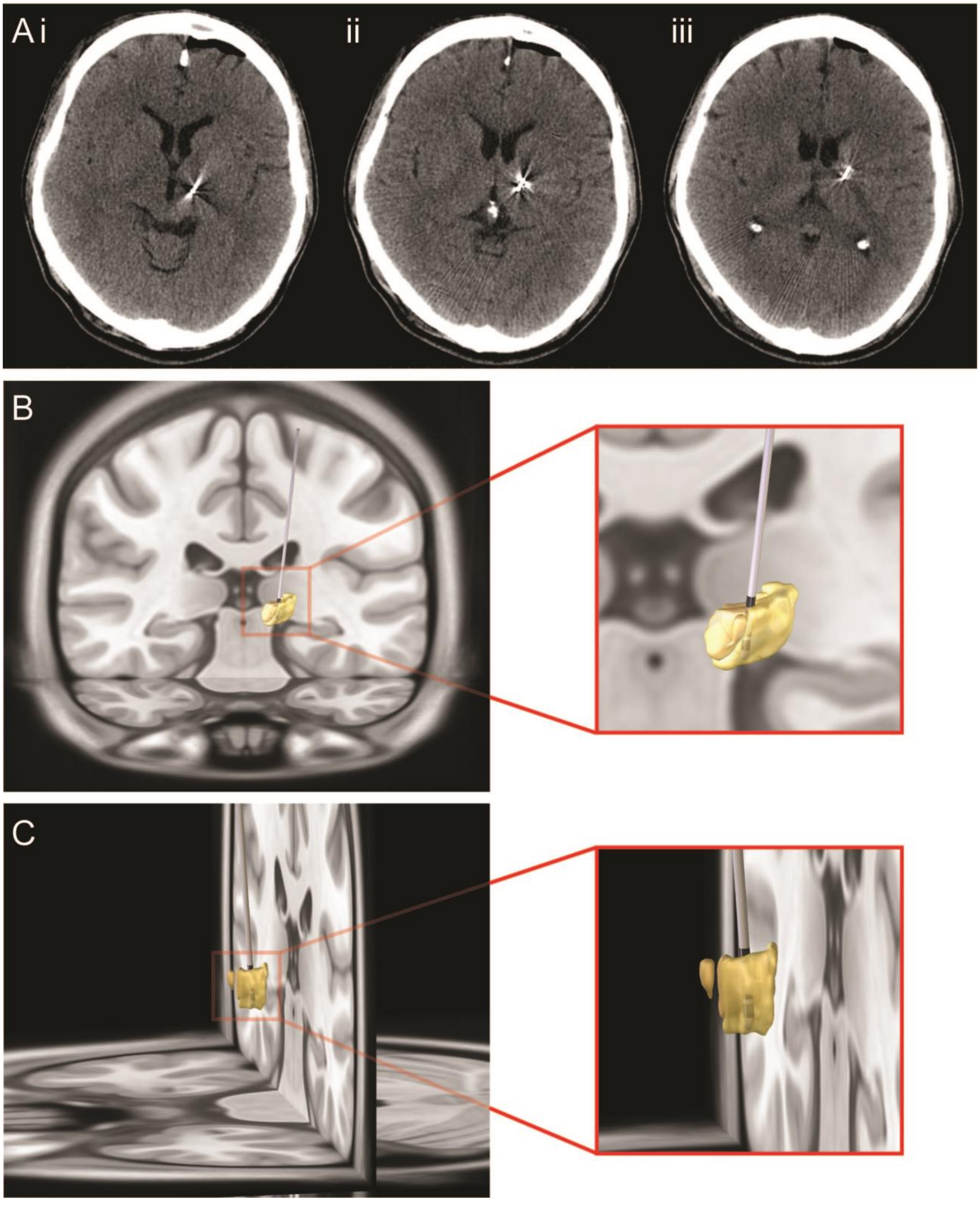

**Supplemental Figure 2: Analysis of peak tremor frequency and power spectra is similar by accelerometry and 3D video-based kinematic analyses; Vim-DBS does not change the frequency of cervical dystonic tremor.** Standard (top two rows) and log-transformed (bottom two rows) power spectra demonstrate similar frequency peaks (inset in each panel) across all conditions; accelerometry and 3D-kinematic analysis produce very similar results. Note differences in units and therefore axes between accelerometry and 3D-kinematics.

# Supplemental Figure 2

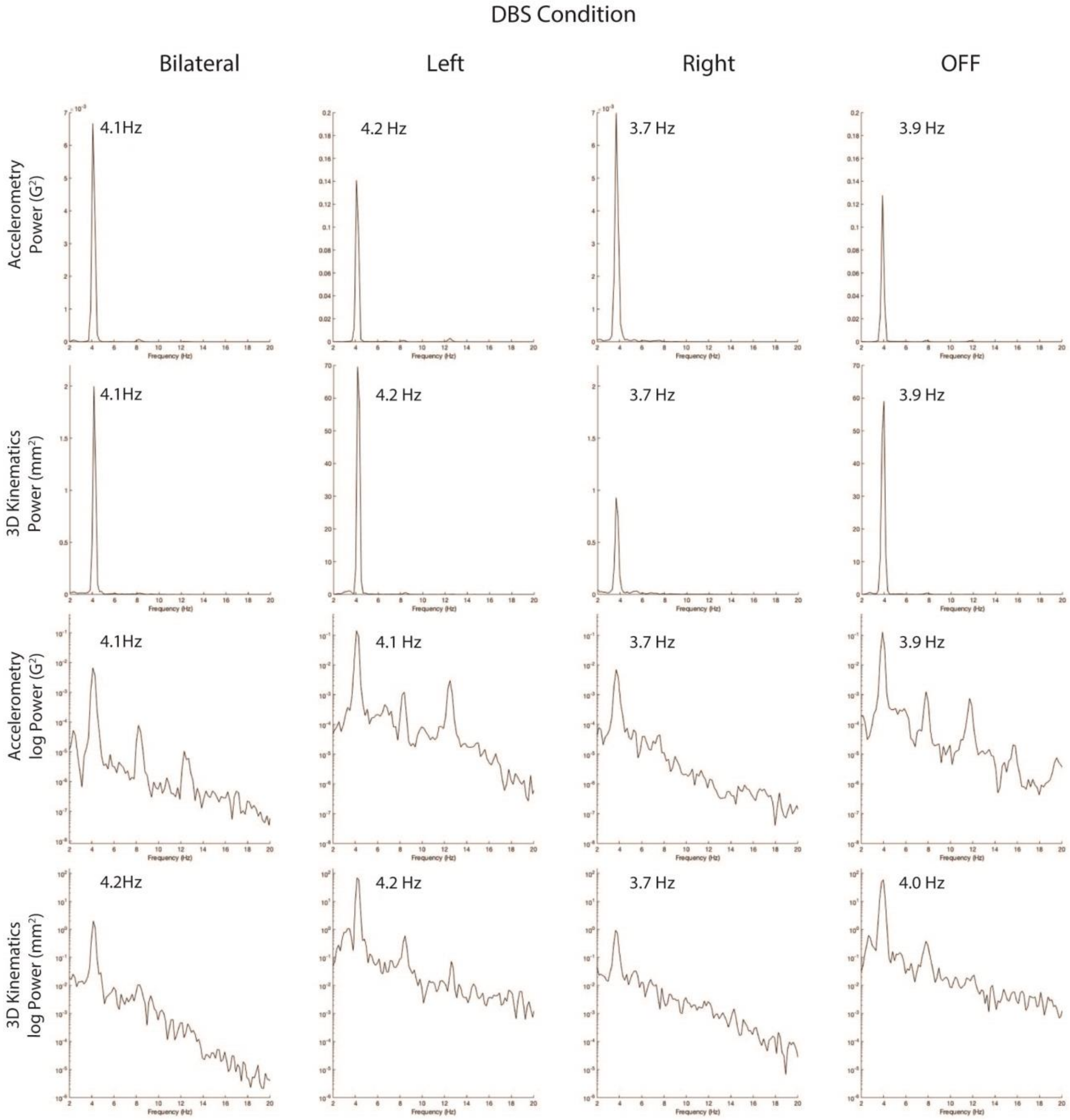

**Supplemental Figure 3: Accelerometry of upper extremity tremor.** With the right Vim-DBS OFF, accelerometry of the left upper extremity shows **(A)** a regular tremor with highest amplitude in the horizontal (medial-lateral) axis. **(B)** Fast Fourier transform shows a sharp peak frequency at 3.7 Hz. **(C)** Cursor position at the measured peak frequency.

Supplemental Figure 3

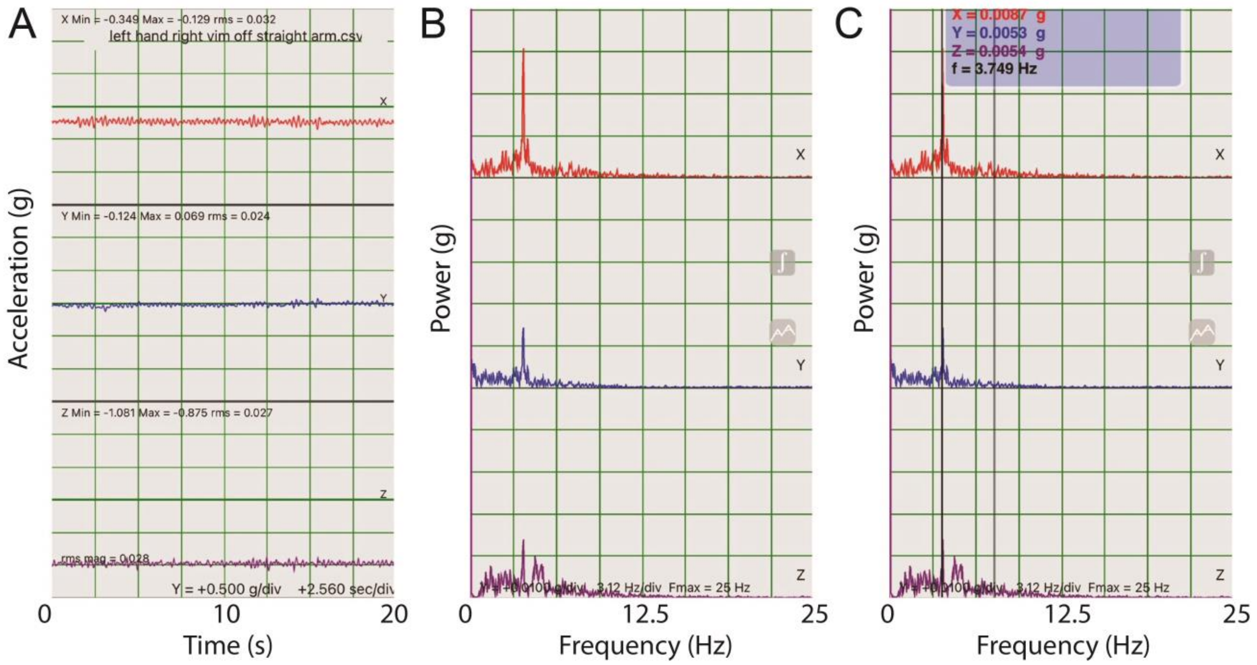

Supplement: Supplemental Figures. — Figures 1–3. [file tohm-12-1-673-s1.pdf]
